# Internet-delivered eye movement desensitization and reprocessing (iEMDR): an open trial

**DOI:** 10.12688/f1000research.2-79.v2

**Published:** 2013-05-07

**Authors:** Jay Spence, Nickolai Titov, Luke Johnston, Blake F Dear, Bethany Wootton, Matthew Terides, Judy Zou

**Affiliations:** 1 Centre for Emotional Health, Department of Psychology, Macquarie University, Sydney, Australia

## Abstract

Recent research indicates internet-delivered cognitive behavioural therapy (iCBT) can reduce symptoms of post traumatic stress disorder (PTSD). This study examined the efficacy of an internet-delivered treatment protocol that combined iCBT and internet-delivered eye movement desensitization and reprocessing (iEMDR), in an uncontrolled trial. Eleven of the 15 participants completed post-treatment questionnaires. Large effect sizes were found from pre-treatment to 3-month follow-up ( *d *= 1.03 – 1.61) on clinician-assessed and self-reported measures of PTSD, anxiety and distress, with moderate effect sizes ( *d* = 0.59 – 0.70) found on measures of depression and disability. At post-treatment, 55% of the participants no longer met criteria for PTSD and this was sustained at follow-up. Symptom worsening occurred in 3 of 15 (20%) of the sample from pre- to post-treatment; however, these participants reported overall symptom improvement by follow-up. Future research directions for iEMDR are discussed.

## Introduction

Results of meta-analyses indicate that both trauma-focused cognitive behavioural therapy (TF-CBT) and eye movement desensitization and reprocessing (EMDR) ^1^ are effective in reducing PTSD symptoms. However, barriers to accessing these treatments include stigma, cost, distance, low mental health literacy, and long waiting lists ^2, 3^.

Internet-delivered psychological treatments may increase access to psychological therapy ^4^. TF-CBT has been delivered via the internet and has shown promise in significantly reducing PTSD symptoms in military personnel ^5, 6^, university students ^7, 8^, and community samples in the U.S. ^7^, Holland ^9^, Iraq ^10^, Australia ^11, 12^ and German-speakers in Europe ^13^. For example, in a previous study ^12^ we evaluated an internet-delivered TF-CBT (iCBT) protocol with Australian adults with a primary diagnosis of PTSD. We found large within-group effect sizes (ESs) and small-to-moderate between-group ESs on measures of PTSD symptoms, depression, anxiety and disability, in a treatment group relative to a control condition.

Time burdens on participants could hinder effective dissemination of internet treatments for PTSD. This study investigated the efficacy of internet-delivered EMDR (iEMDR) on the basis of meta-analytic findings showing that outcomes from face-to-face EMDR are equivalent to TF-CBT with the important distinction that TF-CBT required approximately 23 hours (SD = 11) of homework while EMDR required only 3 hours (SD = 4) ^14^. iEMDR may offer an alternative model of remote treatment for PTSD to iCBT. The present study aimed to explore the acceptability and efficacy of iEMDR when used in conjunction with an iCBT protocol (iCBT/iEMDR course), and evaluated using an open trial design. To increase generalizability of results, inclusion criteria were consistent with those of outpatient services. The primary hypothesis was that the iCBT/iEMDR course would be associated with significant improvements in PTSD symptoms, depression, anxiety, distress, and disability.

Secondary hypotheses were that the treatment would be rated as acceptable to participants and would not be associated with adverse events.

## Methods

The study was approved by the Macquarie University Human Research Ethics Committee (HREC#: 5201100382). Participants provided informed consent. The trial is registered with the Australian and New Zealand Clinical Trials registry as ACTRN12611000151932.

### Participants and recruitment

**Figure 1.**
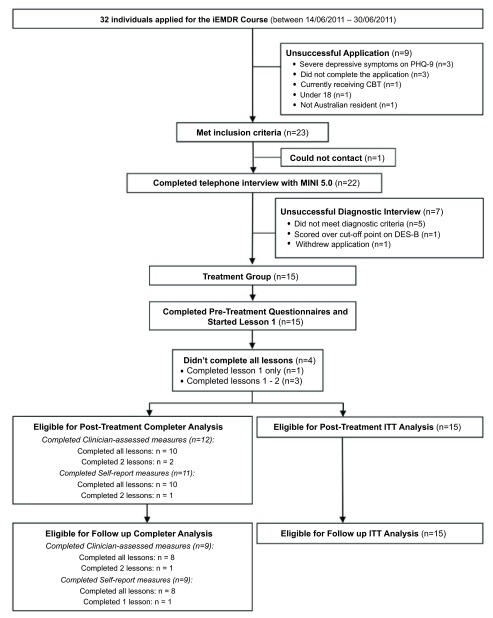
Participant flow chart. iEMDR: Internet-delivered eye movement desensitization and reprocessing. PHQ-9: Patient Health Questionnaire – 9 Item. MINI: MINI International Neuropsychiatric Interview. DES-B: Dissociative Experiences Scale – Brief Version.

Participant flow is shown in Figure 1. Participants were recruited from visitors to a research website that evaluates internet-delivered treatments ( www.ecentreclinic.org). During the recruitment period, which ran over 2 weeks during June 2011, 32 individuals applied and 15 met the following inclusion criteria: (i) self-identified as having a principal complaint of PTSD as indicated by total scores above a clinical cut-off recommended to indicate probable diagnosis of PTSD ^15^ (defined as > 44 on the PTSD Checklist (PCL-C) ^16^) as well as a confirmed primary diagnosis of PTSD determined by clinician-administered interview using the PTSD Symptom Scale-Interview (PSS–I) ^17^; (ii) at least one month had elapsed since the primary trauma; (iii) no psychotherapy for PTSD during the treatment period (however, supportive group and individual counselling that did not specifically target PTSD symptoms was permitted); (iv) if using psychotropic medication, no change in dosage or type of medication 1 month prior to or during treatment; (v) a resident of Australia, (vi) at least 18 years of age, (vii) had computer and internet access, (viii) not *currently* experiencing a psychotic mental illness, extreme current symptoms of depression (defined as a total score > 22 or responding > 2 to Question 9 (suicidal ideation) on the Patient Health Questionnaire - 9 Item (PHQ-9) ^18^, current suicidal intent and plan, or highly dissociative (defined as a total score above 22) on the Dissociative Experiences Scale – Brief Version (DES-B)) ^19^.

### Measures

The primary outcome measures were severity of symptoms of PTSD, measured by the PSS-I and the PCL-C. The PSS-I ^17^ is a 17-item semi-structured clinician-administered interview based on the DSM-IV criteria for PTSD. The PCL-C ^16^ is also a 17-item, self-report scale of PTSD symptoms based on the DSM-IV criteria for PTSD.

Secondary outcomes measures included the Generalized Anxiety Disorder 7-Item Scale (GAD-7, which measures anxiety) ^20^, the PHQ-9 (which measures depression) ^18^, the Mini International Neuropsychiatric Interview (MINI; which was used to determine the presence of a major depressive episode, panic, agoraphobia, social phobia, obsessive compulsive disorder, and generalized anxiety disorder) ^21^, the Kessler 10 Item ^22^ (K10; which measures general distress), and the Sheehan Disability Scale ^23^ (SDS, which measures impairment in psychosocial functioning). Traumatic experiences were assessed using the Life Events Checklist (LEC) ^24^, which provides a list of traumatic events and assesses the occurrence rates of common Criterion A1 (life-threatening) traumas according to the DSM-IV. Additional outcomes included completion rates (percentage of participants who read the six online lessons of the iCBT/iEMDR course within the six weeks of the course), and treatment satisfaction (percentage who reported feeling satisfied with the program or who would recommend it to a friend).

### Intervention

The iCBT/iEMDR course is a six lesson online intervention utilising evidence-based principles of TF-CBT ^25^ and EMDR ^26^. The TF-CBT components were similar to those used in a previous internet-based CBT program for PTSD ^12^. The course comprises text-based information and instructions and educational case stories.

Lesson 1 of the iCBT/iEMDR course includes information about the causes, symptomatology and neurobiology of PTSD, how cognitive, behavioural, and physical symptoms maintain PTSD, and provides instructions for physiological de-arousal strategies. Lesson 2 provides the rationale for using EMDR and detailed instructions about a self-guided iEMDR process. Lesson 3 describes cognitive restructuring strategies. Lesson 4 provides more detail on how to use cognitive restructuring for common trauma-related cognitions. Lesson 5 describes avoidance and safety behaviours and the principles of graded exposure. Lesson 6 describes the principles of relapse prevention.

***iEMDR intervention:*** The EMDR intervention follows the standard EMDR treatment protocol by Shapiro ^26^ with the following adaptations for self-directed use via the internet: the protocol was divided into a desensitization phase (weeks 2–4) followed by a phase aimed at anchoring the positive belief (weeks 5–6). The desensitization phase followed Shapiro’s protocol for reducing the Subjective Units of Distress (SUDS) rating to less than 2. Anchoring the positive belief also followed Shapiro’s protocol ^26^ until the Validity of Cognition (VoC) rating was above 5. Participants were instructed to anchor the positive belief in week 5 of the course only for trauma memories that were no longer distressing (SUDS < 2).

iEMDR was conducted using a web-based EMDR tool ( http://www.rapidtables.com/tool/EMDR.htm). The initial session of EMDR was conducted with the support of the therapist (JS) who guided participants by telephone through the procedure while they accessed the web-based EMDR tool. Further therapist-guided EMDR was provided as requested. Participants who reported not having used self-guided EMDR by mid-treatment were contacted and offered a second guided EMDR session. Instructions for working with blockages to processing were provided in an additional resource one week after giving the iEMDR instructions.

### Therapist

One Clinical Psychologist (JS) provided all clinical contact with participants, which occurred via weekly telephone calls or secure email. The clinician had received Level I and II training in EMDR by a certified EMDR instructor, and had two years experience in administering iCBT and in facilitating EMDR in face-to-face treatment. The clinician was supervised by NT.

### Statistical analysis

Primary analyses were conducted using data only from questionnaire completers, defined as those who completed treatment, post-treatment or follow-up questionnaires. A secondary set of analyses was performed using an intention-to-treat (ITT) model where missing data were addressed by carrying forward the first available data (i.e. baseline-observation-carried-forward model; BOCF).

Pre- to post-treatment and pre-treatment to follow-up changes in questionnaire scores were analysed using paired-sample t-tests. Effect sizes (Cohen’s *d*) ^27^ were calculated based on the pooled standard deviation. All analyses were performed in PASW version 18.0 (SPSS, Inc., Chicago, IL).

Changes in prevalence of PTSD and comorbid disorders were calculated based on the results of telephone administered diagnostic interviews administered at pre-treatment, post-treatment and follow-up.

To measure adverse events we used Tarrier’s ^28^ definitions of treatment worsening, defined as any increase in symptom scores greater than zero from pre- to post-treatment or follow up, and defined serious adverse events as self-reported hospitalizations, suicide attempts, or onset of substance abuse due to treatment.

## Results

### Baseline data

The mean age of participants was 47 years (SD = 10.4), and 10/15 (66%) were women. Ten of 15 participants (67%) reported being either married or in a de facto relationship, 4/15 (27%) reported being separated or widowed and 1/15 (7%) reported being single or never married. Four of fifteen (27%) had a tertiary education, 9/15 (60%) reported having a post-high school certificate and 2/15 (13%) reported as having year 10 high school level education. One participant (7%) was in full-time employment, eight (53%) were employed part-time or studying and six (40%) reported being unemployed, retired, or disabled. Fourteen of fifteen participants (93%) reported having had previous mental health treatment and 10/15 (67%) reported taking medication related to their symptoms of anxiety or depression. One half (5/10) of the participants who completed post-treatment questionnaires reported that they were receiving individual or group supportive counselling during the treatment period that was not specifically directed at the treatment of PTSD symptoms (mean sessions = 3; SD = 2.1). Between post-treatment and follow-up, 25% (2/8) of respondents reported receiving ongoing supportive therapy (not specifically for PTSD) and 13% (1/8) commenced treatment with a psychologist specifically for PTSD (mean sessions = 4; SD = 3.5). There were no reported medication changes during the course. One quarter (2/8) of respondents reported changing their medication post-treatment. Five participants (33%) who reported not having used self-guided EMDR by mid-treatment were contacted and offered a second EMDR session guided by the therapist via telephone. None elected to participate in further EMDR, citing that EMDR had led to an increase in re-experiencing symptoms.

### Trauma history

The most common reported primary trauma was childhood sexual abuse (9/15; 60%), followed by childhood physical abuse (2/15; 13%), domestic violence as an adult (2/15; 13%), witnessing domestic violence as a child (1/15; 7%), captivity (1/15; 7%) and life threatening illness (1/15; 7%). On average, the primary trauma had occurred 32.8 years prior (SD = 12.5). The average age at which the primary trauma occurred was 13.3 years (SD = 12.9). According to the LEC, participants reported having experienced an average of 9.2 types of trauma during their lifetime. The most common was *physical assault* (13/15; 87%), followed by *assault with a weapon* (12/15; 80%), and *other unwanted or uncomfortable sexual experience* (12/15; 80%).

### Attrition

The flow is shown in Figure 1. Eleven participants (73%) completed all six lessons. One participant completed a single lesson and did not complete further assessments. Three participants completed two lessons, one of whom completed post-treatment and follow up assessments and one who completed post-treatment assessments only. One participant completed six lessons, but not the post-treatment or follow-up assessments. Twelve participants completed clinician-assessed post-treatment interviews although one of these participants did not complete the self-report questionnaires. Nine participants completed follow-up questionnaires, including the abovementioned participant who had only completed two lessons. There were no pre-treatment differences between completers and non-completers on the PSS-I, PCL-C or the GAD-7 at pre-treatment.

### Completer analysis

***Primary outcome measures.*** Primary outcome scores for completers improved from pre- to post-treatment as shown in Table 1. Paired-sample t-tests revealed significant reductions on the PSS-I (t _10_ = 3.66, *p* = 0.004) and PCL-C (t _10_ = 2.73, *p* = 0.021) between pre- and post-treatment, and between pre-treatment and follow-up (PSS-I: t _10_ = 4.90, *p* = 0.001; PCL-C: t _10_ = 4.26, *p* = 0.002).

**Table 1.** Descriptive statistics and within-group effects on symptom measures at each assessment.

Measure	Pre-treatment Mean (SD)	Post-treatment Mean (SD)	Follow-up Mean (SD)	Within-group effect size	
				Pre- to post-treatment (95% CI)	Pre-treatment to follow-up (95% CI)
**PSS-I**					
Completers	31.6 (4.7)	19.2 (9.9)	17.1 (8.5)	1.61 (0.65–2.47)*	2.32 (1.16–3.31)*
ITT	31.6 (4.7)	22.0 (9.8)	21.5 (8.6)	1.25 (0.44–2.00)*	1.45 (0.61–2.21)*
**PCL-C**					
Completers	59.0 (11.2)	46.9 (14.9)	43.1 (13.3)	0.95 (0.08–1.76)*	1.33 (0.35–2.22)*
ITT	59.0 (11.2)	50.1 (13.3)	48.1 (11.5)	0.73 (-0.03–1.44)†	0.96 (0.18–1.69)*
**GAD-7**					
Completers	14.1 (4.4)	9.3 (4.7)	8.0 (4.0)	1.06 (0.18–1.87)*	1.42 (0.42–2.31)*
ITT	14.1 (4.4)	11.1 (5.2)	9.9 (3.8)	0.62 (-0.13–1.34)	1.00 (0.22–1.73)*
**PHQ-9**					
Completers	15.3 (4.2)	11.7 (6.2)	11.3 (5.5)	0.70 (-0.14–1.50)*	0.86 (-0.06–1.72)*
ITT	15.3 (4.2)	12.1 (5.3)	11.8 (4.7)	0.66 (-0.10–1.37)	0.78 (0.02–1.50)*
**SDS**					
Completers	21.3 (5.5)	16.6 (10.8)	12.8 (8.9)	0.59 (-0.24–1.39)	1.26 (0.29–2.1)*
ITT	21.3 (5.5)	18.3 (9.9)	16.6 (8.8)	0.37 (-0.36–1.09)	0.65 (-0.1–1.36)†
**K-10**					
Completers	32.2 (5.5)	25.8 (7.3)	24.0 (8.0)	1.03 (0.5–1.84)*	1.28 (0.30–2.16)*
ITT	32.2 (5.5)	27.7 (6.7)	26.9 (6.7)	0.73 (-0.02–1.45)*	0.85 (0.08–1.57)*

*Note:* Intention-to-treat (ITT) model (n=15) was employed with pre-treatment scores carried forward if post-treatment or follow-up data were not available. Completer data were available for 10 participants at post-treatment and 8 at follow-up. Abbreviations: PSS-I: PTSD Symptom Scale – Interview Version; PCL-C: PTSD Checklist – Civilian Version; GAD-7: Generalised Anxiety Disorder 7-Item; PHQ-9: Patient Health Questionnaire – 9 Item; K10: Kessler 10 Item; SDS: Sheehan Disability Scale. * *p* < 0.05; † *p* < 0.06.

***Secondary outcome measures.*** Paired sample t-tests between pre- and post-treatment indicated significant reductions for completers on the PHQ-9 (t _9_ = 2.66, *p* = 0.026), GAD-7 (t _9_ = 2.31, *p* = 0.047), K10 (t _9_ = 2.49, *p* = 0.034), but not on the SDS (t _9_ = 1.66, *p* = 0.131). Significant reductions were reported between pre-treatment and follow-up on the PHQ-9 (t _7_ = 3.13, *p* = 0.017), GAD-7 (t _7_ = 4.16, *p* = 0.004), K10 (t _7_ = 3.95, *p* = 0.006), and SDS (t _7_ = 4.15, *p* = 0.004).

### Intention-to-treat (ITT) analysis

***Primary outcome measures.*** A paired-sample t-test comparing pre- and post-treatment scores for the ITT sample revealed significant reductions on the PSS-I (t _14_ = 3.50, *p* = 0.004), and this was maintained at follow up (t _14_ = 4.59, *p* < 0.0001). Scores on the PCL-C did not significantly improve from pre- to post-treatment (t _14_ = 2.12, *p* = 0.053). However, at follow-up, scores on the PCL-C had significantly improved from pre-treatment (t _14_ = 17.76, *p* < 0.0001).

***Secondary outcome measures.*** Paired sample t-tests for the ITT sample revealed significant reductions between pre- and post-treatment on the K10 (t _14_ = 2.20, *p* = 0.046) but not on the PHQ-9 (t _14_ = 2.12, *p* = 0.053), GAD-7 (t _14_ = 2.02, *p* = 0.063), or SDS (t _14_ = 1.22, *p* = 0.281). There was a significant difference between pre-treatment and follow-up scores on the PHQ-9 (t _14_ = 2.46, *p* = 0.027), GAD-7 (t _14_ = 2.90, *p* = 0.012), K10 (t _14_ = 3.10, *p* = 0.008), but not on the SDS (t _14_ = 2.08, *p* = 0.056).

### Effect sizes

Using the completer analysis, large effect sizes were reported on the PSS-I, PCL-C, GAD-7, and K10 at post-treatment and a moderate effect size was reported on the PHQ-9 and SDS ( Table 1). Large effect sizes were reported on all measures between pre-treatment and follow-up.

Using the ITT analysis, from pre-treatment to post-treatment a large within-group effect size was found on the PSS-I. Moderate within-group effects were found on the GAD-7, PHQ-9, and K10. A small effect size was reported on the SDS. From pre-treatment to follow-up, large effect sizes were found on the PSS-I, PCL-C, and GAD-7, and moderate effect sizes for the PHQ-9, and SDS.

### Clinical significance

Based on the results of the clinician and telephone-administered PSS-I, 6/11 (55%) participants no longer met criteria for PTSD at post-treatment and 5/9 (56%) no longer had PTSD at follow-up. Based on an ITT approach with the BOCF, 5/15 (33%) no longer met criteria for PTSD at post-treatment and follow-up.

With regard to co-morbid diagnoses for completers as measured by clinician-administered MINI, the average number of co-morbid diagnoses reduced from 2.5 (SD = 2.0) at intake to 1.2 (SD = 1.0) at post-treatment, and further reduced to 0.6 (SD = 1.6) at follow-up. According to an ITT analysis the average number of co-morbid diagnoses reduced from 2.5 (SD = 1.7) at intake to 1.4 (SD = 0.9) at post-treatment, and 1.1 (SD = 1.1) at follow-up.

### Adverse events

Three participants reported symptom worsening as defined by Tarrier ^28^ and no participants reported serious adverse events. Of the participants who completed post-treatment questionnaires, three participants showed symptom worsening between pre- and post-treatment on the PCL-C, and one of these had dropped out of treatment after the third lesson. All three improved between post-treatment and follow-up such that no participants worsened between pre-treatment and follow-up. No participants worsened on the PSS-I between any time points.

### Acceptability

At post-treatment, 6/11 (55%) reported that they were *very satisfied* with the course, one participant (9%) was *mostly satisfied*, and 4/11 were *neutral or somewhat satisfied*. None of the participants reported being dissatisfied with the course. Nine of 11 (82%) reported they *would recommend this course to a friend with PTSD*.

EMDR open trial data set, Spence et al. 2013 Raw data (SPSS data set) from the internet-delivered eye movement desensitization and reprocessing (iEMDR) trial.

## Discussion

This study explored the feasibility of a combined iCBT/iEMDR course for treating PTSD in adults using an open-trial design. The results indicated significantly reduced symptoms of PTSD, depression, anxiety, distress, and disability between pre-treatment and three-month follow-up according to an analysis of completers. By post-treatment, 55% of the participants no longer met criteria for PTSD, and the number of comorbid diagnoses had halved. These reductions indicate that PTSD can be treated via the internet using a combination of CBT and EMDR techniques when telephone support from a specialist therapist is also included. With respect to acceptability, this protocol was moderately tolerated, indicating that improvements would be required for further use of this intervention.

Compared with ITT data from our previous trial ^12^, the within-group effect size (ES) on the PCL-C was lower at post-treatment and follow-up. These differences may be due to changes to the protocol, the use of a patient sample composed primarily of childhood sexual abuse survivors, or due to the influence of attrition on the ITT analysis as a result of using a small sample. According to outcomes from the PCL-C, these results compare favourably to other studies that used the same measure with mixed trauma samples in both face to face ^29, 30^ and online interventions ^11, 31^. However, results compared less favourably with face to face TF-CBT treatment of motor vehicle accident survivors ^32^ indicating that outcomes could potentially be improved if future iCBT treatments are tailored and delivered to a specific trauma population.

In our trial, 3/15 (20%) reported symptom worsening according to the PCL-C score between pre- and post-treatment, although all three reported treatment gains by follow-up. Although no serious adverse events (e.g., hospitalizations, suicide attempts, relapse to substance use) occurred during the program, an increase in re-experiencing symptoms (such as intrusive thoughts and increased emotional/physiological reactivity when reminded about the event) following iEDMR lead three participants (20%) to discontinue and five (33%) to cease using iEMDR. This potentially contributed to the higher attrition, moderate acceptability, and limited course and questionnaire completion rates, relative to our earlier study. Although this may have been due to exposure-based components such as iEMDR and *in vivo* exposure, it is important to note that investigations of symptom deterioration and adverse events from the face-to-face literature have failed to indicate differences between exposure- and nonexposure-based treatments ^33^. Furthermore, symptom exacerbation is no higher than reported in waiting lists nor is it greater than the error rates of the instruments used to detect adverse events ^34, 35^.

### Limitations

The absence of a waitlist control condition means that the improvements could have been the results of time, repeated measurement or other non-specific effects. The design did not allow determination of whether the effects were due to the iCBT or iEMDR components. The small sample size composed of a high number of multiply traumatized, childhood sexual abuse survivors may not apply to other PTSD populations.

## Conclusions

The results of this small feasibility study indicate that the combined iCBT/iEMDR protocol is potentially efficacious. The magnitude of gains did not appear to be as large as our previous study, although these may have been attenuated by differences in the sample and iCBT protocol. These results indicate that future research of the relative benefits of iCBT/iEMDR is warranted.
