# Supplementary material for: Internet-delivered eye movement desensitization and reprocessing (iEMDR): an open trial
Source: F1000Res. 2013 May 7;2:79. Originally published 2013 Mar 6. [Version 2] doi: 10.12688/f1000research.2-79.v2 (PMC3901444; doi:10.12688/f1000research.2-79.v2)
Supplement: Peer review history

# Peer review: Referee response for version 2

**DOI:** 10.5256/f1000research.1287.r3963

**Published:** 2014-03-04

**Authors:** Alyssa Boasso

**Affiliations:** 1 Veterans Affairs Medical Center Boston, Boston, MA, USA

I have read this submission. I believe that I have an appropriate level of expertise to confirm that it is of an acceptable scientific standard.

# Peer review: Referee response for version 2

**DOI:** 10.5256/f1000research.1287.r1032

**Published:** 2013-07-01

**Authors:** Jo Abbott

**Affiliations:** 1 Swinburne University of Technology, Hawthorn, Victoria, Australia

The authors’ changes have made have made the paper stronger. I have provided a few minor points of feedback but think the paper acceptable as it is overall, a good feasible evaluation of an innovative online intervention.

The rationale for testing iEMDR is very clear but the rationale for combining iEMDR and i-TF-CBT could be stronger. In the 3 ^rd^ paragraph of the introduction the authors cite evidence that iEMDR is as effective as TF-CBT and can be delivered in a shorter period of time. So this provides a rationale for testing iEMDR but the addition of CBT components does not clearly follow as a rationale. 

As I suggested previously the Klein *et al*. 2010 paper that includes follow-up assessment data is more relevant to cite than the Klein *et al.* 2009 (reference number 11) paper that reports only pre- and post-assessment data for the same trial. There is one sentence under “Adverse events” in the “Results”section that doesn’t seem to clearly follow from the previous sentence: “All three improved between post-treatment and follow-up such that no participants worsened between pre-treatment and follow-up”. However, the changes made to the “Discussion” section about symptoms worsening has made the findings on worsening symptoms much clearer.

I have read this submission. I believe that I have an appropriate level of expertise to confirm that it is of an acceptable scientific standard.

# Peer review: Referee response for version 1

**DOI:** 10.5256/f1000research.309.r857

**Published:** 2013-03-21

**Authors:** Jo Abbott

**Affiliations:** 1 Swinburne University of Technology, Hawthorn, Victoria, Australia

This is an important study in that in that it is exploring the feasibility of an online intervention for PTSD that includes eye movement desensitization as well as CBT. These early stage research studies evaluating novel interventions are important prior to controlled trials and it will be interesting to see how future research can identify the benefits of each component of the intervention.

There were a couple of parts of the flow chart that could be a bit clearer – I too didn't follow where the 16 participants meeting all criteria came from. I also couldn't follow in the flow chart where the following information in the Attrition section fitted: “…one participant completed six lessons, but not the post-treatment assessments”, “Ten participants completed post-treatment questionnaires while eight completed follow-up questionnaires.”

Regarding reference 11 (Klein et al., 2009) – there is a more recent paper about this same trial that includes follow-up data ( Klein et al., 2010) – of relevance given the comparison made to this trial.

I think it is relevant to mention in the discussion that this was a telephone-assisted intervention (not entirely internet-delivered).

I thought that the interpretation of the results would be facilitated if the authors made comment on which of the analyses (ITT or completer) they were placing weight on in drawing their conclusion and why. For example, in the discussion they state that “The results indicated significantly reduced symptoms of PTSD, depression, anxiety, distress, and disability between pre-treatment and three-month follow-up.”, but the disability measure was only significantly different between the groups when using completer analyses.  It would also be useful if Table 1 also noted which of the mean differences were significant (rather than the reader needing to refer back to the text).

I was also interested to know more about the worsening symptoms that some participants reported. Also, the Discussion states that 3/15 participants reported worsening symptoms but in the Results it reads as though five participants stopped using EMDR because they said it “led to an increase in re-experiencing symptoms”.

I have read this submission. I believe that I have an appropriate level of expertise to confirm that it is of an acceptable scientific standard.

# Response

**Published:** 2013-03-24

**Authors:** Jay Spence

**Affiliations:** Macquarie University, Australia

Dear Dr Abbott, Thank you for your feedback. We have addressed your suggestions in an updated manuscript that has recently been uploaded. We would welcome any other edits that you feel are required if they are not adequately addressed in the revised publication. Thank you again for your time in improving this paper. Kind regards, Jay Spence

# Peer review: Referee response for version 1

**DOI:** 10.5256/f1000research.309.r825

**Published:** 2013-03-11

**Authors:** Alyssa Boasso

**Affiliations:** 1 Veterans Affairs Medical Center Boston, Boston, MA, USA

Overall, the writing is succinct and clear. The analyses are appropriate given the study design and the conclusions are justified. There are a few minor areas where further explanation or clarification is needed, these are detailed below. 

- What is the rationale for combining the two therapies? How is iEMDR expected to add to the effectiveness of pre-existing iCBT protocols? 
- The section on recruitment does not explain how 23 people qualified, but only 16 were enrolled. In the iEMDR Intervention subsection, the authors state, “the positive belief” but do not previously define it. Also, how many people were provided special therapist-guided EMDR? Is there conjecture about how additional sessions might have affected outcomes? 
- In the discussion section, the authors state “these results compare favourably to a similar study...” How is the study similar? Clarifying this may help contrast your findings with the following study which used motor vehicle accident survivors. 
- The section on worsening symptoms should address whether other similar studies have encountered the same problem? Also, is there data from the experiment that suggests which aspect of the therapy may be contributing to this issue?

I have read this submission. I believe that I have an appropriate level of expertise to confirm that it is of an acceptable scientific standard.
